# Zithromax^®^ donation for trachoma elimination during the COVID-19 pandemic

**Published:** 2020-09-01

**Authors:** Paul Emerson, PJ Hooper

**Affiliations:** 1 ITI Director: International Trachoma Initiative.; 2 Deputy Director: International Trachoma Initiative.

**Global commitment to the elimination of trachoma remains strong despite COVID-19.**

**Figure:**
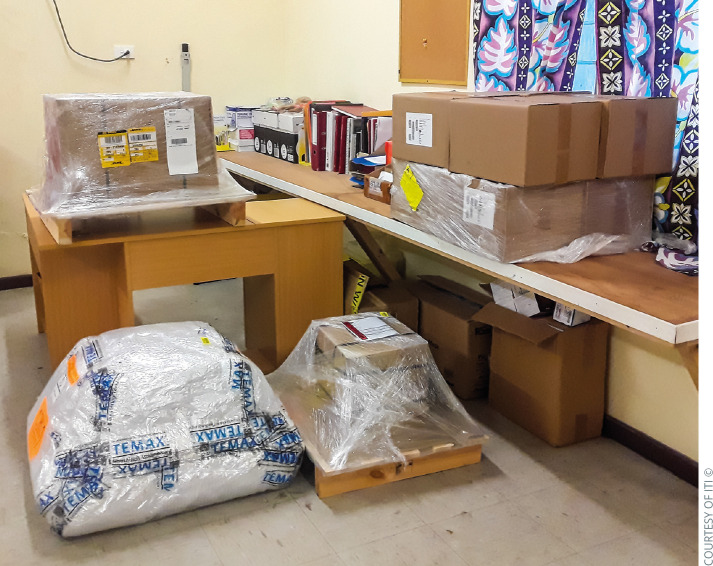
The first shipment of Zithromax^®^ ever to be sent to the island state Nauru, in Micronesia, arrived at their Public Health Eye Mini Dispensary on March 27, 2020.

Following the declaration of COVID-19 as a global pandemic in March 2020 and the subsequent recommendation by WHO that community-based activities for Neglected Tropical Diseases (NTDs) be put on hold (**bit.ly/cov19ntd**), most planned trachoma mass drug administration (MDA) activities have halted around the world. A small number of countries began trachoma MDA in the early months of 2020, but more than 95% of planned MDA activities for trachoma elimination in 2020 have been postponed, or are anticipated to be postponed, in the coming months.

The priorities of all ministries of health across the globe have shifted to focus on the pandemic. NTD personnel in some endemic countries have been redeployed to COVID-19 response activities because of their expertise in at-scale programming and community-based delivery. Because of the effect on programme delivery and human resource allocation, it is currently not possible to predict when countries will be able to safely resume the community-based activities planned for this year; however, ITI and Pfizer’s commitment to a world free from trachoma is unwavering. ITI and its partners stand ready to resume Zithromax^®^ donation shipments and MDA implementation when countries determine that they are ready to restart.

**“These unprecedented circumstances require flexibility and adaptability from country programmes and their donors.”**

Zithromax^®^ availability is not a limiting factor for trachoma interventions in 2020, but disruption to the usual shipment routes may be a challenge in ensuring timely delivery. ITI will need to know when a country plans to resume distributions as soon as the plan is made since air freight availability may be reduced and require more advance notice.

As we have seen, everything is subject to change, but at the moment we don’t anticipate any interruption of supply for trachoma elimination activities in 2021. ITI is forecasting the 2021 demand in the usual way and will process applications for Zithromax^®^ for use in 2021 treatments as normal, using our existing decision-making guidelines.

The process of trachoma elimination is a multi-year commitment. There is not much evidence from the field to suggest that delaying one round of MDA by 6–18 months would have any substantive effect on the elimination timeline, but this may not hold true in areas with high baseline prevalence, in which there is presumably more transmission. Mathematical modelling can be used to determine the risk to the programme as a result of skipping a round of MDA. If such modelling provides evidence of a need for catch-up rounds in certain circumstances, and countries wish to pursue such an approach with their partners, we will consider that in the Zithromax^®^ donation requests. Likewise, if there are interim guidelines from WHO on catch-up rounds or additional targeted treatments in certain circumstances, we would also apply those guidelines when considering Zithromax^®^ donation requests.

These unprecedented circumstances require flexibility and adaptability from country programmes and their donors. ITI and Pfizer are committed to a world free of blinding trachoma, and to working with WHO, countries, and all implementing partners to ensure that the goals of the global elimination programme are met despite this disruption.

